# Demographics, clinical features and outcomes of older patients presenting to an Irish emergency department

**DOI:** 10.1007/s11845-026-04312-3

**Published:** 2026-04-29

**Authors:** Justine Wooding, Kieron Connolly, Angelina Farrelly, Aileen McCabe

**Affiliations:** 1 Department of Emergency Medicine, Tallaght University Hospital, Dublin, D24 Ireland; 2 Department of Geriatric and Acute Medicine, Tallaght University Hospital, Dublin, Ireland; 3 School of Medicine, Trinity College, Dublin, Ireland

**Keywords:** Emergency departments, Frailty

## Abstract

**Background:**

Older adults represent an increasingly significant proportion of Emergency Department (ED) attendances in Ireland, often presenting with complex geriatric syndromes including frailty, cognitive impairment, and multi-morbidity. Tallaght University Hospital (TUH) introduced the Gerontological Emergency Department Intervention (GEDI) team in 2018 to address the needs of this vulnerable cohort through comprehensive geriatric assessment (CGA).

**Aim:**

To characterise the demographics, clinical features, and outcomes of older patients assessed by the GEDI team in TUH ED over a five-year period, and to identify factors independently associated with hospital admission.

**Methods:**

A retrospective observational study was conducted using routinely collected data on all patients assessed by the GEDI team from January 1, 2020 to December 31, 2024. Patient demographics, presenting complaints, CGA components (Rockwood Frailty Score, Barthel Index, 4AT, Mini Nutritional Assessment, SARC-F), ED length of stay, and disposition outcomes were analysed. Multivariate logistic regression identified independent predictors of hospital admission.

**Results:**

Over the study period, 11,256 ED presentations pertaining to 7,055 older patients were assessed by the GEDI team. The mean age was 81.1 years (SD 6.6); 55% were female. The most common presenting complaints were musculoskeletal issues (19.2%) and falls (13.1%). Most patients (64.2%) were moderately frail; 20.6% screened positive for possible delirium. Over one-third were at risk of malnutrition and 48.4% were at risk of sarcopenia. The hospital admission rate was 61.5%. Increasing age, frailty, delirium risk, malnutrition risk, sarcopenia risk, and longer ED stay were independently associated with hospital admission (*p* < 0.05).

**Conclusion:**

This study highlights the clinical complexity of older adults presenting to ED and the utility of interdisciplinary CGA in identifying high-risk features predictive of hospitalisation. These findings support the continued development of integrated geriatric emergency care pathways and reinforce the need for targeted resourcing of geriatric services in Irish EDs.

## Introduction

Within Ireland, the older population comprises of approximately 12.7% of the total population and 25% of patients presenting to the Emergency Department (ED) [1]. Globally there has been an increase in the number of older patients, with predictions that by 2050, 16.7% of the population will constitute patients aged 65 years or more [2]. Tallaght University Hospital (TUH) has seen a 22% increase in older persons attending the ED in the last five years, a pattern that is set to continue over the next 15 years as the local population aged over 75 years increases by 123% or 11,500 people [1].

Older patients often present with complex geriatric syndromes, including falls, cognitive impairment, polypharmacy and frailty [3–6]. Older patients, particularly those presenting with non-specific complaints, are associated with high hospital admission rates, longer hospital length of stay and 30-day mortality rate [7]. Frailty, which is a multifactorial syndrome associated with poor mortality and morbidity outcomes, is a known risk factor for mortality, prolonged hospital admission and functional decline at the time of discharge [1.4].

Understanding the demographics and clinical features of the older population presenting to the Emergency Department is required in order to ensure adequate resourcing of geriatric emergency and communication services. This data analysis may inform evolving models of geriatric emergency assessment and developing ambulatory care pathways.

Tallaght University Hospital introduced a novel interdisciplinary initiative, the Gerontological Emergency Department Intervention (GEDI) team, in mid-December 2018 to meet the needs of a growing frail, older population; and to enhance patient access to the provision of Comprehensive Geriatric Assessment [8]. At the initiation of the service, the GEDI team comprised a clinical nurse specialist, a medical social worker, a physiotherapist, and an occupational therapist. Each healthcare professional was actively involved in frailty screening and the delivery of CGA. Specialist input from a dietitian and a speech and language therapist was available for specific patients, and clinical oversight was provided by a consultant geriatrician and emergency medicine consultants. The team has evolved to include an Advanced Nurse Practitioner and a dedicated geriatric registrar to strengthen embedded senior clinical decision makers within the team and multidisciplinary care planning.

The GEDI service operates Monday to Friday, 07:30 − 18:00, and proactively screens all patients aged ≥ 75 years presenting to the ED via the department’s information system (Symphony). Patients aged ≥ 75 years with high acuity presentations and potential prolonged hospital length of stay are deemed unsuitable for GEDI led comprehensive geriatric assessment. Stable patients (defined as those patients with an Irish National Early Warning System (INEWS) (score of two or less) are reviewed using the PRISMA-7 frailty screening tool [9, 10]. Patients who do not screen positive for frailty and who the emergency clinicians deem likely not to benefit from acute gerontological input, are typically not seen by the GEDI service. Patients who screen positive for frailty are flagged for CGA to be done the GEDI service within working hours. The CGA done by the GEDI service assesses functional status, cognition, nutrition, mobility, social supports, and medical complexity. Results and recommendations are documented in a structured electronic health record proforma, and are verbally communicated to the overseeing emergency medicine clinician.

This study sought to characterise the demographics, clinical features and outcomes of patients assessed by the Gerontological Emergency Department Intervention Team (GEDI) in Tallaght University Hospital Emergency Department.

## Methods

### Setting and design

This study is a retrospective single-centre study analysing data inputted by the GEDI team into the TUH Emergency Department information system between 1st January 2020 and 31st December 2024. The project was classified by the TUH/SJH research ethics committee (project ID: 4857) as a clinical audit and was approved to proceed. No funding was sought for this study. The STROBE standardised reporting guidelines were followed to ensure the standardised conduct and reporting of the study [11] (Supplementary File 2).

Tallaght University Hospital is an Irish level 4 model hospital which is also a designated trauma and hyperacute FAST+ receiving hospital in the local region. Tallaght University Hospital Emergency Department sees patients aged 16 years and older.

### Study eligibility

Patients were included in the study if they had a completed comprehensive geriatric assessment by a member of the GEDI team within the study period. ED patients aged 75 years and older were the key inclusion criteria for assessment by the GEDI team. Patients younger than 75 years were assessed by the GEDI team if identified by the emergency medicine team as potentially benefitting from a GEDI assessment. The GEDI service typically worked between 07:30 and 18:00 Monday to Friday during the study period. Patients not assessed by GEDI were excluded from this study.

### Data collection

The GEDI team exclusively use what is colloquially known as a “GEDI screening tool” on the Emergency Department Information System (Symphony, EMIS Health). The GEDI team use the tool on patients who they deem may be potentially suitable for a comprehensive assessment by the team. A data report was abstracted from all patients screened by the GEDI team between 1st January 2020 and 31st December 2024. The following patient specific data was collected: age, gender, arrival mode to hospital, referral source, and primary presenting complaint and disposition outcomes. ED length of stay was collated as a key performance indicator.

### Comprehensive geriatric assessment variables

Data related to the components of the comprehensive geriatric assessments such as the Rockwood frailty assessment, Barthel index assessment, 4AT assessment, mini nutritional assessment and sarcopenia assessment were also collated.

Frailty was assessed using the Rockwood Clinical Frailty Scale (CFS). This scoring scale assesses patients between 1 (very fit) to 9 (terminally ill) [12]. The GEDI team additionally groups patients into three categories, robust (score 1–3), mildly frail or frail (score 4–6) and severely frail (score *≥* 7).

The Barthel Index was used to assess activities of daily living (ADLs) with a maximum score indicating complete independence and the minimum score indicating total dependence [13]. Scores can additionally be grouped into three categories, high dependency (scores 0 to 11), mild dependency (scores 12 to 17) and low dependency (scores *≥* 18).

The 4AT score is an assessment tool for detection of delirium or cognitive impairment [14]. Patients can be categorised into delirium or cognitive impairment unlikely (score 0), possible cognitive impairment (score 1 to 3) and possible delirium and/or cognitive impairment (score *≥* 4) [14].

The mini nutritional assessment assess patients at risk of malnutrition and patients can be categorised as normal or well-nourished (scores *≥* 12), at risk of malnutrition (scores 8 to 11) and malnourished (scores *≤* 7) [15].

The SARC-F assessment assess the risk of sarcopenia, or age-related muscle wasting, and patients can be categorised as normal (scores 0 to 3) and at risk of sarcopenia (score *≥* 4) [16].

### Statistical analysis

Descriptive statistics were performed. Data are expressed as mean for normally distributed continuous data and count (%) for discrete data.

Pearson’s and Spearman’s correlation tests were used to determine the strength of association between various variables included in the dataset, particularly variables that were likely to be included in regression models including, age, frailty and delirium variables. Variables that were strongly correlated (Pearson or Spearman R = > 0.6) with each other were not used in the regression analysis. When developing a regression model, all equations had the variables age and gender included. Variables that were determined significantly associated with admission to hospital were chosen first, followed by variables that were trending toward significance. Other variables were also considered, as sample size allowed, including variables highlighted in the literature and specific variables that could be altered via interventions.

Multi-regression bivariate logistic analysis was used to determine the independent association of multiple secondary outcomes with admission to hospital. Multi-regression bivariate logistic analysis was complete using the whole data set. Prior to the development of regression models to determine independently associated factors with admission to hospital, the multi-collinearity of variables needed to be considered.

## Results

282,512 patients presented to the Emergency Department at Tallaght University Hospital between 1st January 2020 and 31st December 2024. In the study period, a “GEDI screening tool” was documented on 11,256 presentations pertaining to 7,055 patients in TUH ED.

### Demographics

The mean age of the included study cohort was 81.12 years (range of 16 to 107 years; SD 6.6). There were 6189 female (55%) and 5067 male (45%).

The majority of patients (*n* = 5,927, 52.7%) presented by ambulance followed by motor vehicle (*n* = 4,763, 42.3%) (Table 1). Most patients (*n* = 7,935, 70.5%) self-presented to Tallaght University Hospital ED. GP referrals pertained to 21.3% (*n* = 2,392) of the cohort and 3.8% (*n* = 433) were transferred from a nursing home or residential care facility (Table 1).

**Table 1:** Demographics and disposition outcomes of patients assessed by the GEDI team in Tallaght University Hospital Emergency Department

Demographics	Total presentations	Percentage (% of total presentations 11,256)
*Gender*		
Female	6189	55
Male	5067	45
*Arrival Mode*		
Ambulance	5927	52.7
Helicopter	6	0.1
Motor vehicle	4763	42.3
Motorbike	1	0.0
Taxi	92	0.8
Public transport	40	0.4
Walk	82	0.7
Garda	4	0.0
Other	325	2.9
Unknown	16	0.1
*Referral source*		
Self	7935	70.5
General practitioner	2393	21.3
Nursing home or Residential care	433	3.8
Other Hospital	141	1.1
Outpatient Department or Clinic	208	2.0
Public Health Nurse	11	0.1
Gardai	5	0.0
Emergency Department	130	1.2
*Disposition outcome*		
Admitted	6927	61.5
Discharged	4225	37.5
Home with no follow-up	858	7.6
GP follow-up	1643	14.6
Outpatient or Clinic follow-up	1167	10.4
Did not wait	75	0.7
Refused Treatment and/or Admission	6	0.1
Other	476	4.2
Transferred to another hospital	80	0.7
Transferred to the mortuary	21	0.2
No available data	3	0.0

### Presenting complaint as categorised by Manchester Triage

There were multiple various presenting complaints with the most prominent being musculoskeletal problems (*n* = 2,158, 19.2%), followed by generally unwell (*n* = 1,644, 14.6%) fall or collapse (*n* = 1,471, 13.1%) and respiratory problems (*n* = 1,211, 10.8%) (Table 2).

**Table 2:** Broad presenting complaints of patients assessed by the GEDI Team

Presenting complaints	Total presentations	Percentage (% of total presentations 11,256)
Musculoskeletal problem	2158	19.2
Generally unwell	1644	14.6
Fall or collapse	1471	13.1
Respiratory problem	1211	10.8
Gastrointestinal problem	1110	9.9
Other	771	6.8
Trauma	745	6.6
Cardiovascular problem	701	6.2
Genitourinary problem	471	4.2
Bleeding	281	2.5
Neurological problem	177	1.6
Local infection or abscess	92	0.8
Facial problem	91	0.8
Eye problem	52	0.5
Dermatology problem	44	0.4
Behaving strangely	31	0.3
Psychiatric problem	26	0.2
Sepsis	24	0.2
Diabetic problem	23	0.2
Ear problem	18	0.2
Overdose or poisoning	18	0.2
Foreign body	12	0.1
Sore throat	12	0.1
Allergy	4	0.0
Dental problem	4	0.0
No available data	65	0.6

### Variables of the comprehensive geriatric assessment

#### Rockwood frailty assessment

82.2% (*n* = 9,251) had a completed Rockwood frailty assessment with the majority scoring within the mildly frail or frail category (*n* = 5,939, 64.2%), followed by 21.8% within the robust category (*n* = 2,013) and 14.0% within the severely frail category (*n* = 1,299). The mean score was 4.84 (range of 1 to 9; SD 1.533) which falls within the mildly frail or frail category (Table 3).

**Table 3:** Variables of the comprehensive geriatric assessment for patients assessed by the Gerontological Emergency Department Intervention (GEDI) Team in Tallaght University Hospital Emergency Department

Individual-levels variables	*N*	Percentage	Mean	Minimum	Maximum	SD
*Rockwood Frailty Assessment*	9251	82.2	4.84	1	9	1.533
Robust (score 1–3)	2013	21.8				
Mildly frail or Frail (score 4–6)	5939	64.2				
Severely frail (score ≥ 7)	1299	14.0				
Rockwood Frailty Assessment score	9251	82.2				
Barthel Index score	7662	68.1	16.39	0	20	4.517
*4AT Delirium Assessment*	6615	58.8	1.66	0	12	2.630
Delirium or cognitive impairment unlikely (score 0)	3661	55.3				
Possible cognitive impairment (score 1–3)	1593	24.1				
Possible delirium and/or cognitive impairment (score ≥ 4)	1361	20.6				
4AT Delirium Assessment Score	6615	58.8				
*Mini Nutritional Assessment*	7462	66.3	10.72	0	14	2.965
Malnutrition (score ≤ 7)	1138	15.3				
Risk of malnutrition (score 8–11)	2761	37.0				
Normal nutrition (score ≥ 12)	3563	47.7				
Mini Nutritional Assessment score	7462	66.3				
*SARC-F Assessment*	7017	62.1	3.61	0	10	2.651
Normal (score 0–3)	3624	51.6				
At risk of sarcopenia (score ≥ 4)	3393	48.4				
SARC-F Assessment score	7017	62.3				

#### Barthel index

68.1% (*n* = 7,662) had a completed Barthel Index with a mean score of 16.39 (range 0 to 20; SD 4.517). This indicates that the majority of patients were mildly dependent in terms of their ADLs (Table 3).

#### 4AT delirium assessment

58.8% (*n* = 6,615) had a completed 4AT delirium assessment with 55.3% (*n* = 3,661) falling into the category of delirium or cognitive impairment unlikely, followed by 24.1% (*n* = 1,593) in the category of possible cognitive impairment and 20.6% (*n* = 1,361) within the category of possible delirium and/or cognitive impairment. The mean score was 1.7 (SD 2.6) and this falls into the category of possible cognitive impairment (Table 3).

#### Mini nutritional assessment

66.3% (*n* = 7,462) had a completed mini nutritional assessment with 47.7% (*n* = 3,563) being well nourished, followed by 37.0% (*n* = 2,761) being at risk of malnutrition and 15.3% (*n* = 1,138) being classified as malnourished. The mean score was 10.72 (SD ± 3) which falls within the category of at risk of malnutrition (Table 3).

#### Sarcopenia assessment

62.3% (*n* = 7,017) had a completed SARC-F assessment with 51.6% (*n* = 3,624) being categorised as normal and 48.4% (*n* = 3,393) being categorised as at risk of sarcopenia. The mean score was 3.61 (SD 2.7) which just falls within the normal category (Table 3).

### Outcomes

The majority of the study population of older patients (61.5%, *n* = 6,927) presenting to TUH Emergency Department were admitted, with 37.5% (*n* = 4,225) older patients discharged. Of the patients discharged home, the majority (*n* = 1,643, 14.6%) were discharged with GP follow-up, followed by discharge to an outpatient or clinic follow-up (*n* = 1,167, 10.4%). Eighty (0.7%) (*n* = 80) were transferred to another hospital while twenty-one (0.2%) died. The mean ED length of stay was 17.28 h (SD 137.888).

### Strength of association

The degree of frailty (increasing frailty), risk of delirium, declining nutritional status and risk of sarcopenia were all strongly associated (*p*-value < 0.001) with admission to hospital (Table 4).

**Table 4:** Chi-square tests of strength of association between admission to hospital and individual-level variables

Individual-levels variables	Admission to Hospital		
	Value	df	Asymptomatic significance (2-sided)
Degree of frailty (CFS)	176.096	1	< 0.001
Risk of Delirium (4AT)	309.076	1	< 0.001
Declining Nutritional status (MNA)	332.663	1	< 0.001
Risk of Sarcopenia (SARC-F)	188.531	1	< 0.001

Increasing age (*p*-value < 0.001), increased total time within the department (*p*-value < 0.001), increasing frailty (*p*-value 0.003), risk of Delirium (*p*-value < 0.001), declining nutritional status (*p*-value < 0.001) and risk of sarcopenia (*p*-value 0.011) were all found to be independently associated with admission to hospital (Table 5).

## Discussion

This study provides a detailed characterisation of older adults assessed by the GEDI team at Tallaght University Hospital (TUH) over a five-year period. With over 11,000 presentations and more than 7,000 individual patients assessed, this study reflects the complexity of older patients presenting to an Irish ED.

A key finding of this study is the high hospital admission rate among the GEDI-assessed cohort (61.5%). High admission rates in older adults is consistent with previous literature highlighting the complexity and acute care needs of older adults presenting to the Emergency Department (ED) [3]. The admission rate in the population assessed by the GEDI service is higher than overall average conversion rate of 52% for patients aged 75 years year to date in Tallaght University Hospital ED in 2025. This is likely due to GEDI service prioritising patients who screening positive for frailty. It is also notable that there has been a negative 6.3% conversion rate in the admission rate in the over 75 year old population since 2022 to our ED despite a 43% rise in ED attendances in the same population over the same time period [17]. Local initiatives such as development of a dedicated Age-related acute medical unit, with a Registered Advanced Nurse Practitioner (RANP) in Gerontology have indicated positive impact on flow of patients aged 75+ [18].

The majority of patients presented via ambulance and self-referred, underscoring the urgent and often unplanned nature of their presentations. Musculoskeletal complaints and falls were the most common presenting complaints, reflecting recognised geriatric syndromes such as functional decline, and supporting the rationale for early multidisciplinary assessment [6, 7].

The mean Rockwood Clinical Frailty Score of 4.8 situates the study cohort within the ‘mildly frail or frail’ category. Patients in the GEDI cohort are generally pre-selected based on positive screening on PRISMA-7 so may reflect a selection bias. Frailty was found to be independently associated with hospital admission (*p* = 0.003), reinforcing the value of structured frailty assessment within the ED. This is particularly relevant in the Irish healthcare context, where demand for hospital beds is high and efficient care pathways for frail older adults are increasingly necessary. Of note our findings are consistent with those of the Prevalence of Frailty in European Emergency Departments (FEED) international flash mob study which included five Irish sites and found that 40% of older people using European emergency care had CFS 5 + [19].

Among patients who underwent a Comprehensive Geriatric Assessment (CGA), 21.8% were classified as robust (CFS 1–3). This is notable given that many ED frailty services primarily focus on patients already exhibiting moderate to severe frailty. The inclusion of robust older adults within the GEDI model reflects a broader, more preventative approach to geriatric emergency care. Such inclusive screening aligns with emerging consensus that early identification, even among robust patients, can support anticipatory care planning and reduce the risk of deterioration [20].

The finding that 20.6% of patients screened positive for possible delirium is clinically significant and is notably higher than the prevalence of 13% seen in the subgroup analysis of the 361 Irish patients included in the European Geriatric Emergency Departments Registry Study [21]. This is likely to reflect the high burden of cognitive vulnerability in the older emergency population seen by the Tallaght University Hospital GEDI service. Delirium is now well established as a predictor of adverse outcomes including increased mortality, longer hospitalisation, institutionalisation, and long-term cognitive decline [22, 23]. The 4AT screening tool has been validated in emergency and acute care settings as a rapid, reliable method for delirium detection and has been recommended for routine use due to its brevity and accuracy [14]. Integrating delirium screening into GEDI workflows facilitates early recognition and appropriate multidisciplinary management. Recent studies underscore the importance of embedding cognitive assessments within CGA processes to inform disposition planning and reduce avoidable complications [24].

The Barthel Index further indicated that most patients were mildly dependent in activities of daily living (mean score 16.4), while assessments using the 4AT tool showed that nearly one-fifth had possible delirium or cognitive impairment. Older adults often present with atypical or subtle symptoms which can delay diagnosis and the initiation of treatment. Early recognition of delirium is important as it can indicate clinical deterioration and is associated with increased rates of mortality. These findings are significant as both functional decline and cognitive impairment have been shown to predict poorer ED and inpatient outcomes, including increased length of stay and readmission risk [25].

Nutritional status and sarcopenia were also prevalent concerns in this population. Nutritional status is an important factor within the older population as it can have an influence on frailty and cognitive functions. Over half of those assessed were either malnourished or at risk of malnutrition. These findings are similar to an historical longitudinal cohort study conducted in France over a 7-year period, which also found that 37% of patients were considered to be at risk of malnutrition. Both malnutrition and sarcopenia were found to be significantly associated with hospital admission (*p* < 0.001 and *p* = 0.011, respectively), which echoes international evidence and supports the inclusion of nutritional and sarcopenia screening as standard components of ED-based comprehensive geriatric assessments [15, 16].

Importantly, this study confirms that increasing age, frailty, delirium risk, malnutrition, and sarcopenia independently predict hospital admission. These findings strengthen the integration of geriatric models of care into the acute setting and align with national policy goals under the HSE’s National Clinical Programme for Older People and Sláintecare. The high burden of geriatric syndromes observed in this cohort underscores the importance of structured CGA and interdisciplinary input in emergency settings. Expansion of ambulatory frailty pathways, hospital-at-home models, and geriatric-led decision making may reduce admissions and enhance community-based care [26, 27].

The high prevalence of frailty, cognitive impairment, malnutrition, and sarcopenia points to key areas for targeted intervention. GEDI assessments frequently result in referrals to rapid access day hospitals, medication and nutrition reviews, community frailty teams, and activation of home care supports. Embedding frailty flags within the electronic health record further aids in continuity and risk stratification at future presentations [17]. Linking CGA findings to specific actions is critical to improving outcomes and avoiding unnecessary admissions [28, 29].

The mean ED length of stay in the study cohort was notably long (17.3 h). This mean length of stay is not consistent with Health Service Executive’s target key performance indicators (KPIs) for EDs. Reducing ED length of stay and boarding is critical. Whilst this study was not specifically designed to look at any correlation with length of stay and outcome such as mortality in our population, systematic review by Lauque et al. has shown an association between ED length of stay and in house mortality [30]. A number of factors could be considered to account for the prolonged ED length of stay. Firstly, Tallaght University Hospital’s 495 adult beds has been deemed insufficient to serve its rapidly growing population of over 700,000 people living in its catchment area. The Irish government has committed to increase 196 new beds for Tallaght University Hospital [31]. Secondly, the prolonged ED length of stay may also reflect both the complex needs of the cohort and systemic challenges in accessing inpatient and/or community services. Tallaght University Hospital has prioritised integrated care in its latest strategic report [32]. Finally, the prolonged length of stay may also reflect the time-intensive nature of comprehensive geriatric assessment and interdisciplinary care assessment and coordination particularly for presentations such silver trauma.

### Study strengths and limitations

This study has several strengths, including its large sample size, multi-year dataset, and use of validated assessment tools. However, it is limited by its retrospective design and the single-centre setting, which may affect generalisability. Data completeness varied across assessment tools, and some patients did not receive all assessments, which could introduce selection bias.

Another potential source of bias relates to relatively small study cohort compared to the overall number of patients who presented to the ED in the study period. The study cohort consists of those patients assessed by the GEDI service. The number of patients assessed by the GEDI service are limited by the operational considerations. Since its inception, the GEDI service is currently only resourced to cover Monday to Friday and contingent on having a fully staffed roster which has at times has been intermittently challenged with recruitment issues. The service has always prioritises assessing those patients who would most likely benefit from their service, namely patients who have been identified as frail. The authors acknowledge that these caveats are important to consider in the interpretation of the study’s results.

Lastly, the study does not assess longer-term outcomes such as readmission, functional recovery, or mortality, which would be valuable for evaluating the full impact of GEDI interventions.

## Conclusion

This study demonstrates that frailty, cognitive impairment, nutritional risk, and sarcopenia are highly prevalent and independently associated with hospital admission among older adults attending the ED and who were seen by the GEDI service. These findings reinforce the policy relevance of the GEDI model within the evolving landscape of Irish health reform. The high burden of frailty and complex geriatric syndromes among older ED attendees directly aligns with strategic objectives under the HSE’s Integrated Care Programme for Older Persons (ICPOP), which aims to deliver more coordinated, anticipatory care for older people across hospital and community interfaces [27]. Similarly, the Enhanced Community Care (ECC) programme promotes development of specialist community teams to manage frailty closer to home and reduce avoidable ED attendances [33]. The outcomes observed in this study suggest that structured ED-based CGA, as delivered by GEDI, may complement these national programmes by identifying at-risk individuals early and activating downstream supports. Future expansion and evaluation of GEDI should be considered in conjunction with ICPOP and ECC rollout.

## Data Availability

Data is available upon request to the corresponding author.
